# Differentiation through E‐nose and GC‐FID data modeling of rosé sparkling wines elaborated via traditional and Charmat methods

**DOI:** 10.1002/jsfa.13178

**Published:** 2024-01-05

**Authors:** Raquel Muñoz‐Castells, Margherita Modesti, Jaime Moreno‐García, María Rodríguez‐Moreno, Alexandro Catini, Rosamaria Capuano, Corrado Di Natale, Andrea Bellincontro, Juan Moreno

**Affiliations:** ^1^ Department of Agricultural Chemistry, Edaphology and Microbiology, Marie Curie (C3) and Severo Ochoa (C6) Buildings, Agrifood Campus of International Excellence ceiA3 University of Córdoba Córdoba Spain; ^2^ Department for Innovation of Biological, Agrofood and Forest Systems (DIBAF) University of Tuscia Viterbo Italy; ^3^ Department of Electronic Engineering University of Rome Tor Vergata Rome Italy

**Keywords:** chemometric analysis, electronic nose, PLS‐DA, sparkling wine, volatile compounds

## Abstract

**BACKGROUND:**

The growing demand for rosé sparkling wine has led to an increase in its production. Traditional or Charmat wine‐making influence the aromatic profiles in wine. An analysis such as gas chromatography makes an accurate assessment of wines based on volatile detection but is resource intensive. On the other hand, the electronic nose (E‐nose) has emerged as a versatile tool, offering rapid, cost‐effective discrimination of wines, and contributing insights into quality and production processes because of its aptitude to perform a global aromatic pattern evaluation. In the present study, rosé sparkling wines were produced using both methods and major volatile compounds and polyols were measured. Wines were tested by E‐nose and predictive modelling was performed to distinguish them.

**RESULTS:**

Volatile profiles showed differences between Charmat and traditional methods, especially at 5 months of aging. A partial least square discriminant analysis (PLS‐DA) was carried out on E‐nose detections, obtaining a model that describes 94% of the variability, separating samples in different clusters and correctly identifying different classes. The differences derived from PLS‐DA clustering agree with the results obtained by gas‐chromatography. Moreover, a principal components regression model was built to verify the ability of the E‐nose to non‐destructively predict the amount of different volatiles analyzed.

**CONCLUSION:**

Production methods of Rosé sparkling wine affect the final wine aroma profiles as a result of the differences in terms of volatiles. The PLS‐DA of the data obtained with E‐nose reveals that distinguishing between Charmat and traditional methods is possible. Moreover, predictive models using gas chromatography‐flame ionization detection analysis and E‐nose highlight the possibility of fast and efficient prediction of volatiles from the E‐nose. © 2023 The Authors. *Journal of The Science of Food and Agriculture* published by John Wiley & Sons Ltd on behalf of Society of Chemical Industry.

## INTRODUCTION

The production of rosé sparkling wine has experienced significant growth in recent years because of its increasing popularity and high global consumer demand.^1^ This production process involves the use of different grape varieties, associated with the edaphoclimatic conditions of each region.^2^, ^3^ One of the grape varieties commonly used to produce rosé sparkling wines is Garnacha tintorera, also known as Alicante Bouschet. It is a *teinturier* grape variety, known for its intense color and aroma.^4^ This variety is cultivated in various regions around the world, including Spain, France, Italy, Australia and California, where the warm climate conditions are ideal for growing Garnacha grapes. The wines made from this variety are typically dry in style, with a broad and rich palate featuring watermelon, strawberry and lemon flavors.^5^ Although the grape variety is important for this type of wine, the defining characteristic of sparkling wines lies in the presence of carbon dioxide (CO_2_), which is produced in a second fermentation of the base wine that takes place in sealed containers.^6^ There are two main methods of sparkling wine production: the traditional method, also known as the Champenoise or the classic method, and the Granvas, bulk or Charmat‐Martinotti method (commonly referred as Charmat). The main difference between the two methods is that, in the traditional one, the second fermentation takes place in sealed bottles, whereas, in the Charmat method, it is carried out in an isobaric tank.^3^ However, when comparing the traditional and Charmat methods, there are additional differences between the processes besides the vessel in which it takes place: (i) the volume of the container where the second fermentation takes place (i.e. small‐volume glass bottles for the traditional method and larger isobaric tank in the Charmat method), which influence the volume of the base wine in contact with lees during aging; (ii) the material of the containers (glass for the traditional and stainless steel for the Charmat method); (iii) maturation in contact with lees, being generally longer (months or years) in the traditional method and shorter (usually limited to 6 months) in the Charmat method; (iv) the wine in the traditional method cannot be filtered, only clarified, whereas the Charmat method allows for both possibilities; and (v) the Charmat process involves faster and more cost‐effective production techniques compared to the traditional, which is more complex and expensive. These differences lead to wines having significantly different quality traits: the wine obtained via the traditional method generally acquires more complex sensory attributes because of the longer aging on lees and the larger volume of base wine in contact with it. By contrast, the Charmat wine is characterized by fresher and fruitier characteristics.^2^, ^3^, ^7^, ^8^, ^9^, ^10^

The differences attributed to the method used during secondary fermentation in the final wine have been disputed. These differences were overestimated because the main characteristics of the wine occurred before secondary fermentation.^3^, ^11^ However, it is well established that, during the second fermentation, the yeasts are subjected to stressful conditions such as low pH, nutrient depletion, CO_2_ overpressure and high ethanol concentrations.^3^ Under these conditions, yeast metabolism produces different volatile and non‐volatile compounds.^8^, ^12^, ^13^ Moreover, during aging on lees, the production of volatiles and non‐volatile compounds becomes even more relevant as a result of autophagic/autolytic processes. From a chemical perspective, these aging‐related reactions can lead to significant variations in the volatile profiles of wines, thereby impacting their sensory quality.^9^, ^10^, ^14^, ^15^, ^16^, ^17^ Moreover, in this type of wine, physical properties such as foam, effervescence and color also play a crucial role.^8^, ^18^ These complex characteristics may be significantly attributed to the use of different yeast strains. To date, only a few studies are available on the aromatic characteristics of sparkling wines made with the two different methods using the same base wine, yeasts and conditions. In this context, the wine aromatic characterization is not an easy task because of the high complexity and heterogeneity of its headspace. As a result of the intricate complexity of wine headspace composition, human evaluation remains the most superior method for assessing its quality. However, the language employed by expert wine tasters often possesses an air of mystery and can be more poetic than precise.^19^ The evaluation of wine quality through human sensory analysis is valuable but subjective. Instrumental methods such as gas chromatography (GC) are precise but expensive and labor‐intensive and are referred to volatile compounds detection, but not to aromatic pattern evaluation, which is affected not just by the presence of the volatile molecule. In this context, the electronic nose (E‐nose) offers great operational flexibility and is especially useful in differentiating wines based on their aromatic fingerprint. For example, the E‐nose has been employed to (i) discriminate wines from different grape varieties, vintages, or grown under diverse conditions^20^, ^21^; (ii) identify off‐flavors caused by undesirable microorganisms^22^; (iii) assess wine quality and differentiate sweet wines obtained through partial fermentation or no fermentation of the grape must^23^, ^24^; and (iv) discriminate wines from the same grape variety grown in different areas.^25^ In this regard, E‐nose using fast GC such as the Heracles E‐nose (Alpha MOS, Toulouse, France) has been used for the discrimination of wines by mean of the volatile profiles obtained.^26^, ^27^ The use of E‐noses provides a promising and efficient alternative to traditional sensory analysis, offering valuable insights into wine quality and production processes.^28^

The present study aimed to evaluate the differences in the aromatic profile of rosé sparkling wines made from the Garnacha tintorera grape variety using both the traditional and Charmat methods starting from the same base wine, yeasts and conditions. Additionally, the study aimed to compare the potential of the E‐nose and GC‐flame ionization detection (GC‐FID) as a discriminating tool using rosé sparkling wines as a case study.

## MATERIALS AND METHODS

### Wines and winemaking conditions

#### Grape must

Garnacha tintorera grapes were harvested from Tarazona de la Mancha (Albacete, Spain) within the Protected Designation of Origin ‘La Manchuela’ on 8 September 2021, yielding a harvest of 200 kg. The grapes were destemmed and crushed, resulting in 150 L of must with a density of 1083 g L^−1^, pH of 3.5 and total acidity of 6.52 g L^−1^.

#### Base wine

A base wine was produced from the must, following the vinification process at Pago Puerto Carretas (Villaviciosa, Córdoba, Spain). The grape must was fermented with a dose of 10 g L^−1^ Primaflora VR (AEB, Stuttgart, Germany) and 20 g L^−1^Glutaferm One (AEB) active dry yeasts, corresponding to 10^6^ and 2 × 10^6^ viable cells, respectively. The alcoholic fermentation took place in a 200‐L tank at 20 °C for 11 days, reaching a relative density value of 0.9827. The analytical profile of the resulting wine was: ethanol content 11.4% v/v, malic acid 1.62 g L^−1^, total acidity 7.72 g L^−1^, volatile acidity 0.32 g L^−1^ and reducing sugars 0.41 g L^−1^. At the end of alcoholic fermentation, 0.1 g L^−1^ potassium metabisulphite and 1 g L^−1^ dry lactic acid bacteria Extraflore Pure Fruit from Enotecnia (Barcelona, Spain) were added for malolactic fermentation.

The base wine to obtain the rosé sparkling wine was prepared by adding 24 g L^−1^ sucrose, 550 mg L^−1^ diammonium phosphate, 100 mg L^−1^ mannoproteins (Super Bouquet MN, Agrovin), 30 mg L^−1^ bentonite and dipotassium meta‐bisulphite (K_2_S_2_O_5_) to reach a final concentration of 50 mg L^−1^ in total sulphur dioxide (SO_2_) content. This base wine was inoculated with 3 × 10^6^ cells of PDM *Saccharomyces cerevisiae* active dry yeast (Agrovin), previously conditioned in accordance with the provider's recommendations, for the second fermentation in sealed recipients.

#### Sparkling wine production

##### Traditional method

To obtain the sparkling wine by the traditional method, 45 bottles (each with a volume of 750 mL) were filled with the same base wine. The bottles were sealed with a plastic lid and a metal crown cap. They were then stored horizontally in a conditioned chamber at a constant temperature of 12 ± 1 °C and 60–75% humidity until the second fermentation was completed. An aphrometer coupled to a control bottle was used to monitor the pressure inside the bottles. After 5 months from the beginning of the second fermentation, two bottles were riddled, disgorged and corked to carry out the analyses, and the same processes were repeated also after 11 months from the beginning of the second fermentation.

##### Charmat method

The second fermentation to the production of rosé sparkling wine using the Charmat method was carried out in a 36‐L food‐grade steel autoclave, at a constant temperature of 12 °C for 5 months. During this period, the internal pressure was monitored using an external manometer attached to the autoclave. At the end of the experiment and previous to wine bottling under isobaric conditions, the temperature of the tank was lowered to −2 °C for 10 days to stabilize the wine. The wine was then bottled using an isobaric filler, once it had stabilized and all the lees had settled to the bottom of the tank. Two bottles were used for analysis and the remainder were stored for 11 months in the same conditioned chamber used for the bottles of the traditional method to repeat the analysis.

### Analytical methods

The oenological parameters ethanol, titratable acidity, volatile acidity, pH and relative density at 20 °C were determined following the protocols established by the International Organization of Vine and Wine.^29^ Absorbances at 280, 420, 520 and 620 nm were measured using an Cary 60 UV‐visible spectrophotometer (Agilent Technologies, Santa Clara, CA, USA).

Major volatile compounds and polyols were analyzed by gas chromatography on an Agilent 6890 GC system (Agilent Technologies) provided with a flame ionization detector (FID) and a capillary column CP‐WAX 57 CB (60 m; 0.25 mm; 0.4 μm film thickness). The analysis was performed by direct injection of wine samples following the methodology of Peinado *et al*.^30^ For this, a mixture of 10 mL of wine and 1 mL of a 1.018 g L^−1^ solution of 4‐methyl‐2‐pentanol (CAS 108‐11‐2), as internal standard, and 0.2 g of solid calcium carbonate was sonicated for 30 s, followed by centrifugation at 3800*g* for 10 min at 2 °C. Then, 0.7 μL of the supernatant was injected into the inlet of the GC system. The absolute quantification of methanol, higher alcohols (1‐propanol, isobutanol, 2‐methyl and 3‐methyl‐1‐butanol, and 2‐phenyl ethanol), acetaldehyde, acetoin, ethyl acetate, ethyl lactate, diethyl succinate, and the polyols glycerol and 2,3‐butanediol (*levo* and *meso* forms) was carried out using calibration tables established with standard solutions of known concentrations, which were subjects to the same treatment as the wine samples. Chemical compounds for the standard solutions were provided by Sigma‐Aldrich (St Louis, MO, USA). All analyses were performed in triplicate.

### Electronic nose

The electronic nose (E‐nose) used was designed, developed and assembled at the University of Rome Tor Vergata and is based on an array of 12 quartz microbalances (QMBs). In these sensors, small changes in mass (Δ*m*) on the absorbing layer of the quartz surface lead to a shift in frequency (Δ*f*) in the electrical output signal of the oscillator circuit. Within a range of minor alterations, the change in frequency (Δ*f*) is directly proportional to the change in mass (Δ*m*). The chosen QMBs are made from AT‐cut quartz crystals with a fundamental frequency of 20 MHz, corresponding to a mass resolution of a few nanograms.^31^

The QMBs were functionalized by seven metal complexes (Mg, Co, Cu, Zn, Fe, Mn and Sn), free base (H2) of 5, 10, 15, 20‐tetrakis‐(4‐butyloxyphenyl) porphyrin (TBPP), free base (H3), copper, phosphorus and manganese complexes of 5,10,15‐triphenylcorrole (TPC),^28^ deposited on the quartz surface by spray casting. The sensing molecules were synthesized in the Department of Chemical Science and Technology at the University of Rome Tor Vergata. These molecules were fully characterized for their sensitivity to volatile organic compounds.^32^, ^33^, ^34^, ^35^ Each QMB is individually linked to an oscillator circuit. A temperature‐compensated quartz crystal is used as a reference for measuring oscillator output frequencies, providing a frequency resolution of 0.1 Hz. The system also includes temperature and relative humidity sensors. Gas delivery is managed through a tubeless embedded pneumatic system that incorporates a poly(methyl methacrylate) manifold with two inlets and one outlet. This system is connected to a miniature diaphragm pump (flow range 0–200 sccm), a three‐way electronic valve, a proportional electronic valve and a flow sensor. The instrument is connected and powered via a USB connection. The acquisition of data, instrument functions and settings are all controlled using proprietary software developed in Matlab.^31^

Wine measurement was carried out as follows: 10 mL of wine was incubated in 25‐mL closed vials (equipped with silicon septum) at 30 °C for 20 min. Then, the equilibrated headspace was extracted for 90 s using a stream of filtered air and delivered into the electronic nose sensor cell. After each measurement, a pure air stream was used to clean the E‐nose for an additional 300 s, establishing the reference signal. Sensor signals were calculated as the resonant frequency shift between the two steady conditions corresponding to sensors exposed to pure air and the sample. The ensemble of sensor signals is composed of patterns (fingerprints) encoding the global composition of the headspace.

### Statistical analysis

Data were analyzed using different statistical software packages. Analysis of variance was carried out with the Centurion XVI (Statgraphics, The Plains, VA, USA) package to establish significant differences between the five wines studied. E‐nose numerical data were pre‐treated with two normalization filters (mean centering and autoscaling) and then analyzed with regression analysis (partial least square discriminant analysis; PLS‐DA) using the venetian‐blind as a validation method (blind thickness = 1). For the construction of the model, three latent variables were selected, reaching a cumulative percentage of explained variability of 94.33%. The E‐nose data were also used to build a linear regression model using a principal components regression with the E‐nose data as independent variables (predictor variables) and major volatile compounds as dependent variables (response variables). Both sets of data were first autoscaled and venetian blinds with blind thickness = 1 was used as cross‐validation method. Three principal components were used to reach a cumulative percentage of explained variability of 96.76%. All the multivariate analyses were performed using Matlab R2013a (MathWorks, Natick, MA, USA) and PLS Toolbox (Eigenvector Research, Inc., Manson, WA, USA).

## RESULTS AND DISCUSSION

### Oenological variables

**Figure 1:**
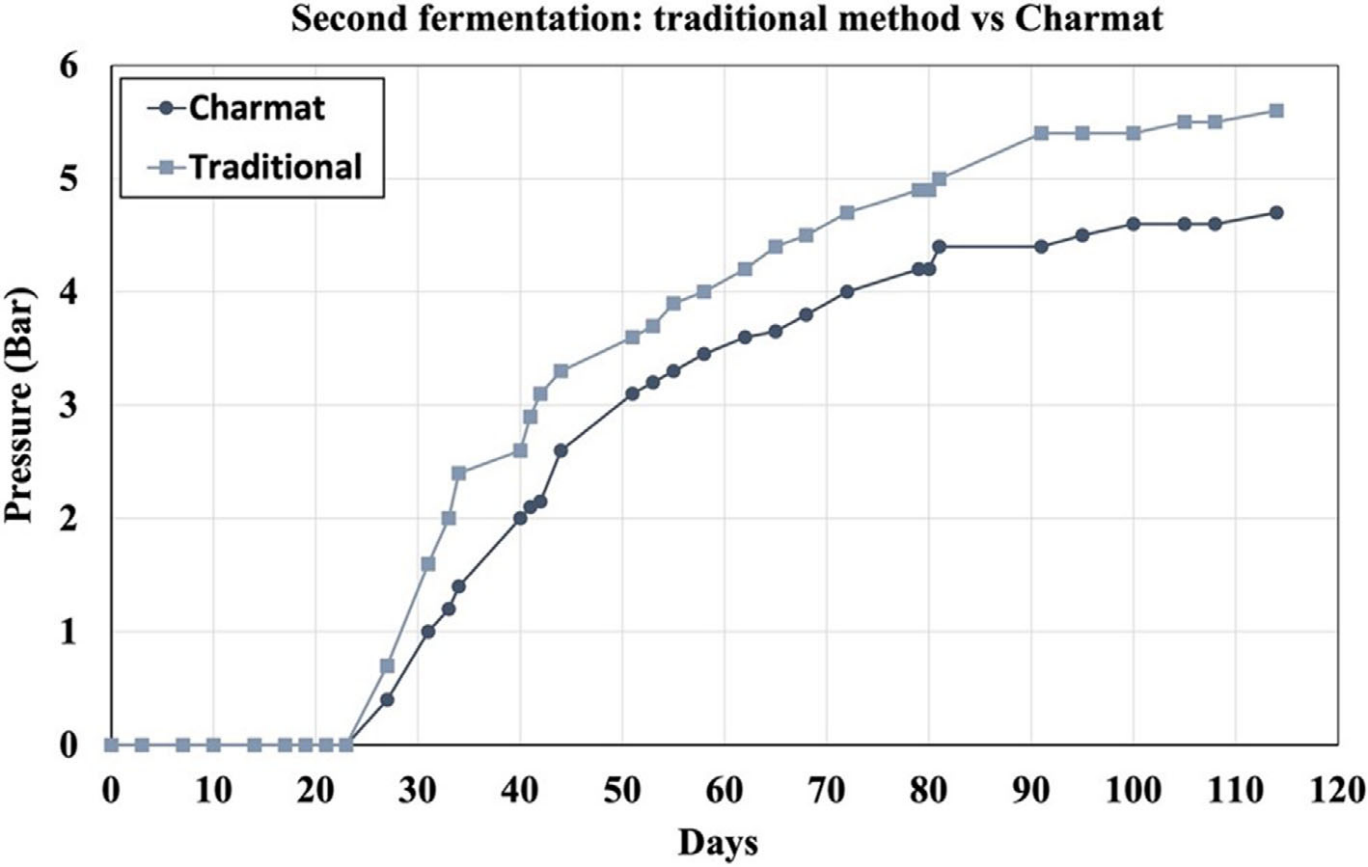
Evolution of the internal pressure in the second fermentation of a base wine by traditional and Charmat methods.

Figure 1 shows the changes in terms of pressure in both the traditional and Charmat methods. The yeasts requires an adaptation period to the base wine for initiatating the second fermentation, which is 27 days when the pressure of CO2 gas released reaches a value measurable by the aphrometer. The traditional method resulted in higher pressures compared to the Charmat method, reaching 5.6 bar and 5 bar respectively by day 114. These differences can be mainly attributed to the small differences in the headspace volume of the vessel where the second fermentation was carried out. The volume and homogenization of the base wine can also influence the viability and fermentation activity of the yeasts responsible for the second fermentation process.^3^ All wines obtained at the end of the experiments meet the standards required to be classified as natural sparkling wines, exceeding the 3.5 bar at 20 °C proposed by the International Organisation of Vine and Wine.^29^

The results obtained for the oenological variables of the base wine and the sparkling wines coming from the traditional or the Charmat methods are shown in Table 1.

**Table 1:** Mean ± SD and homogeneous groups of the oenological variables of wines

Parameter	BW	Trad5M	Charmat5M	Trad11M	Charmat11M
Relative density	0.9827 ± 0.0007 ^d^	0.966 ± 0.003 ^b^	0.961 ± 0.004 ^a^	0.9711 ± 0.0007 ^c^	0.9691 ± 0.0003 ^bc^
pH	3.18 ± 0.03 ^a^	3.57 ± 0.00 ^b^	3.56 ± 0.06 ^b^	3.57 ± 0.00 ^b^	3.57 ± 0.00 ^b^
Volatile acidity (g L^−1^)	0.317 ± 0.006 ^a^	0.65 ± 0.05 ^c^	0.70 ± 0.00 ^d^	0.58 ± 0.02 ^b^	0.59 ± 0.00 ^b^
Total acidity (g L^−1^)	7.72 ± 0.00 ^d^	6.0 ± 0.2 ^a^	5.90 ± 0.06 ^a^	6.34 ± 0.06 ^b^	6.76 ± 0.04 ^c^
Ethanol (% V/V)	11.4 ± 0.1 ^a^	12.4 ± 0.2 ^c^	12.0 ± 0.1 ^b^	12.3 ± 0.1 ^c^	12.1 ± 0.1 ^b^
Absorbance 420 nm	1.600 ± 0.007 ^b^	0.877 ± 0.002 ^a^	0.960 ± 0.002^a^	0.845 ± 0.001^a^	0.9880 ± 0.0002 ^a^
Absorbance 520 nm	2.206 ± 0.008 ^c^	1.080 ± 0.001 ^b^	1.166 ± 0.003 ^b^	1.027 ± 0.003 ^ab^	1.184 ± 0.001 ^a^
Absorbance 620 nm	0.351 ± 0.004 ^ab^	0.2298 ± 0.0004 ^a^	0.259 ± 0.001 ^a^	0.212 ± 0.001 ^a^	0.2610 ± 0.0004 ^b^
TPI (absorbance 280 nm)	37.63 ± 0.04 ^d^	17.64 ± 0.02 ^a^	18.52 ± 0.08 ^b^	19.5 ± 0.2 ^c^	19.62 ± 0.03 ^c^

Different lowercase letters in the same row indicate different homogeneous groups at a significance level of 0.05. Wine samples: BW, base wine; Trad5M, traditional method analyzed at 5 months of aging; Charmat5M, Charmat method at 5 months of aging; Trad11M, traditional method at 11 months aging on the lees; Charmat11M, Charmat method with 5 months aging and bottled for 6 months. TPI: total polyphenols index

The lowest pH was found in the base wine sample, with a value of 3.18. After the second fermentation, the pH increased in the sparkling wine, and no significant differences were found between the two methods. Therefore, the pH remained stable from 5 to 11 months, when the traditional wine remained on the lees, whereas the Charmat wine had already been bottled. Volatile acidity also increased in the sparkling wines. After 5 months from the second fermentation, the traditional sparkling wine showed lower acetic acid concentrations than the Charmat one. However, after 11 months, these values decreased and became equal, indicating that the yeasts in the traditional method no longer contributed additional acidity to the wine. Both traditional and Charmat wines had lower total acidity after 5 months compared to 11 months, increasing by 0.34 and 0.86 g L^−1^, respectively. These increases can be explained by a better integration of the CO_2_ gas dissolved in the sparkling wine during this period, which increases the difficulty of its complete elimination before the analysis of the total acidity. The ethanol content ranged between 12.4 and 12% (v/v), being lower in the wine produced by the Charmat method and maintained over time. The relative density measured in all the wines (Table 1) after the second fermentation shows values between 0.961 and 0.971, indicating that all of them are dry wines with a residual sugar content of less than 5 g L^−1^. No significant differences were observed between the two methods for absorbances at 420, 520 and 620 nm. The total polyphenols index (TPI) showed a slight increase with the aging on lees and storage time in the traditional and Charmat methods, respectively.

### Major volatile compounds and polyols

Table 2 shows the content of major volatile compounds and polyols after 5 months from the second fermentation and after 11 months when the traditional wine remained on the lees, whereas the Charmat was already bottled without lees. The wines obtained by the two methods showed significant differences in their volatile profiles because all the compounds showed two or more homogeneous groups.

**Table 2:** Mean and standard deviations of major volatile compounds and polyols expressed as mg L^−1^ quantified by GC‐FID

Compound	BW	Trad5M	Charmat5M	Trad11M	Charmat11M	HG
Acetaldehyde	96 ± 5 ^a^	140 ± 13 ^c^	113 ± 4 ^b^	132 ± 10 ^c^	143 ± 8 ^c^	3
1,1‐Diethoxyethane	20.5 ± 0.8 ^b^	0 ^a^	0 ^a^	0 ^a^	0 ^a^	2
Acetoin	63 ± 3 ^a^	78 ± 4 ^b^	65 ± 4 ^a^	65 ± 4 ^a^	97 ± 7 ^c^	3
Methanol	59 ± 2 ^a^	109 ± 10 ^bc^	100 ± 9 ^b^	117.4 ± 7.0 ^cd^	126 ± 5 ^d^	4
1‐Propanol	37.8 ± 0.6 ^a^	39 ± 1 ^ab^	41 ± 2 ^b^	43.4 ± 0.6 ^c^	44 ± 2 ^c^	3
Isobutanol	38.3 ± 0.2 ^b^	35 ± 1 ^a^	38.8 ± 0.3 ^b^	39 ± 1 ^b^	39 ± 1 ^b^	2
2‐Methyl‐1‐butanol	48.9 ± 0.5 ^b^	46 ± 1 ^a^	48.6 ± 0.5 ^b^	50 ± 1 ^b^	51 ± 2 ^b^	2
3‐Methyl‐1‐butanol	297 ± 3 ^b^	268 ± 5 ^a^	300 ± 2 ^b^	298 ± 9 ^b^	301 ± 7 ^b^	2
2‐Phenylethanol	45 ± 4 ^bc^	54 ± 2 ^d^	39.1 ± 0.8 ^a^	41 ± 3 ^ab^	49 ± 3 ^c^	4
Ethyl acetate	42 ± 1 ^a^	69 ± 2 ^b^	77.7 ± 0.3 ^c^	78 ± 2 ^c^	98 ± 8 ^d^	4
Ethyl lactate	50 ± 3 ^a^	126 ± 7 ^c^	112 ± 6 ^b^	149 ± 5 ^d^	152 ± 5 ^d^	4
Diethyl succinate	8.1 ± 0.5 ^b^	9.1 ± 0.9 ^bc^	5.8 ± 0.4 ^a^	11 ± 1 ^c^	8.0 ± 0.8 ^b^	3
2,3‐Butanediol *levo*	337 ± 17 ^a^	730 ± 66 ^e^	404 ± 18 ^b^	584 ± 25 ^c^	659 ± 13 ^d^	5
2,3‐Butanediol *meso*	69 ± 8 ^a^	248 ± 18 ^d^	137 ± 6 ^b^	225 ± 14 ^cd^	218 ± 14 ^c^	4
Glycerol (g L^−1^)	6.56 ± 0.07 ^a^	15 ± 1 ^c^	7.2 ± 0.4 ^a^	12 ± 2 ^b^	13.2 ± 0.6 ^b^	3

Different lowercase letters in the same row indicate different homogeneous groups at a significance level of 0.05. Wine samples: BW, base wine; Trad5M, traditional method analyzed at 5 months of aging; Charmat5M, Charmat method at 5 months of aging; Trad11M, traditional method at 11 months aging on the lees; Charmat11M, Charmat method with 5 months aging and 6 months bottled.

Carbonylic compounds such as acetaldehyde showed differences between Charmat and traditional methods at 5 months. However, their concentrations reached similar levels over time, even though the Charmat was no longer in contact with the lees. Among the six alcohols quantified, only isobutanol, 2‐methyl‐1‐butanol and 3‐methyl‐1‐butanol showed two homogeneous groups, with 1‐propanol three and methanol and 2‐phenylethanol showing four homogeneous groups. The traditional wine at 5 months stands out from the others, presenting lower concentrations of these compounds. However, after 11 months, this wine showed no significant differences compared to the Charmat.

The concentrations of 1‐propanol increase over time both in the traditional wine in contact with the lees and in the bottled Charmat. This suggests that the lees have little influence on this increase because there are no significant differences between the two wines. The same is true for ethyl esters, where ethyl acetate and ethyl lactate concentrations also increase after 11 months. This finding is in agreement with a previous study reporting higher concentrations of ethyl esters in traditional sparkling wines compared to the Charmat,^7^ as well as observed in the present study. Ethyl esters increase over time, with diethyl succinate being one of the main esters during fermentation and aging on the lees.^7^, ^11^

The polyols 2,3‐butanediol (in its *levo* and *meso* forms) and glycerol reach their highest contents in the wine from the traditional method at 5 months, where the ratio volume of wine to yeast lees is lower than in the wine from Charmat. Although 2,3‐butanediol has been associated with aging on the lees^9^, ^16^ its concentration decreased over time in this study.

### E‐nose data

**Figure 2:**
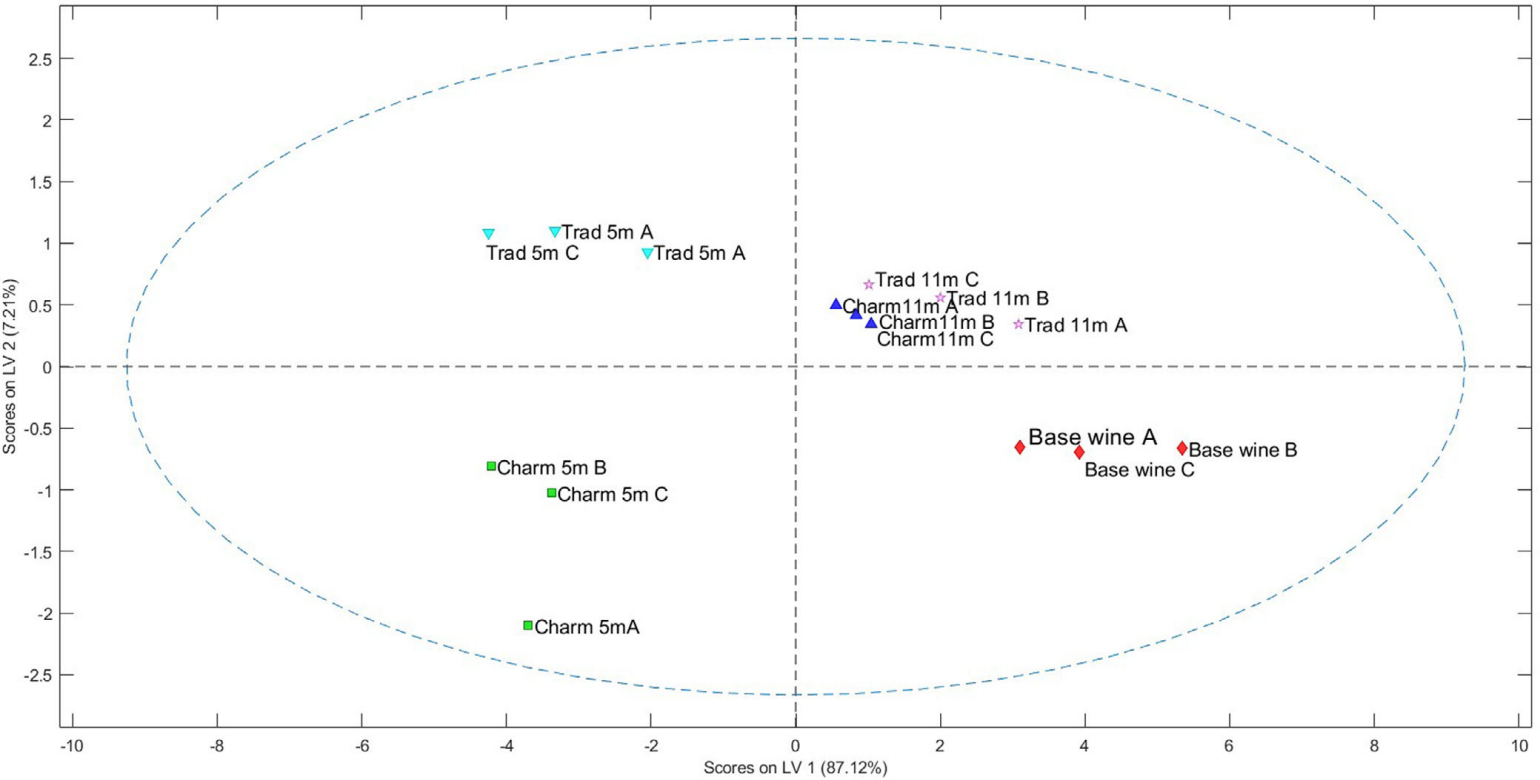
Score plot of latent variables (LV1 *versus* LV2) of the partial least square discriminant analysis model built with pre‐treated E‐nose data of the sparkling wines. Wine samples: base wine. Trad5M A, B and C: traditional method analyzed at 5 months of ageing. Charmat5M A, B and C: Charmat method at 5 months of aging. Trad11M A, B and C: traditional method at 11 months aging on the lees. Charmat11M A, B and C: Charmat method with 5 months aging and 6 months bottled.

**Figure 3:**
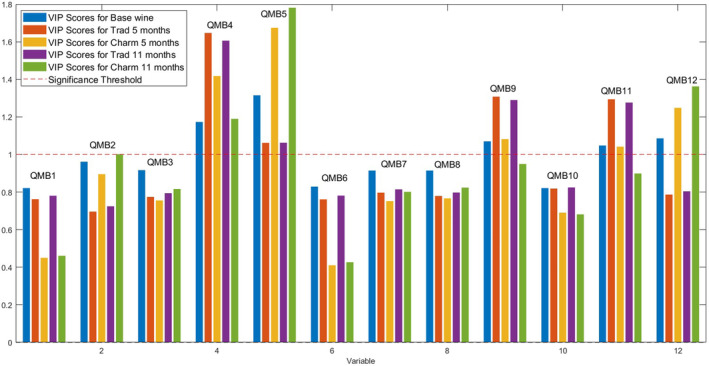
Effect of variables [loadings, quartz microbalances (QMB) nose sensors] on the pattern recognition discrimination of the partial least square discriminant analysis performed on the acquisitions of the electronic nose headspaces samples of different wines. The influences are evaluated as a projection of the variables of importance in projection (VIP) generated by each variable (QMBs) and appreciated on the basis of an efficacy threshold (equal to 1).

When molecules are adsorbed in the coated layer above the surface of the E‐nose QMBs, there is a corresponding change in their oscillation frequency. This change is directly proportional to the amount of mass absorbed, and the resulting alteration in the electric signal is then quantified. The data from the sensor array are typically analyzed using advanced statistical methods such as principal component analysis or PLS‐DA, which transforms the initial sensor signals into new variables formed by linear combinations of these signals. These newly derived variables can be graphed on a two‐dimensional plot known as a score plot. These PLS‐DA score plots are generally interpreted with the assumption that the distance between points represents the similarity between samples. Consequently, clusters observed in the score plots are interpreted as groups of similar samples.^28^ Figures 2 and 3 show, respectively, the PLS‐DA score plot and the plot related to the influence of the different loadings (the 12 different sensors) on the selected latent variables.

The obtained model describes about 94% of the variability on the first two latent variables (LV1 87.12% and LV2 7.21%). From the score plot, it is possible to identify four different clusters. A first cluster includes the base wine, two separate clusters with the two wines from the two production methods after 5 months (i.e. Charm A, B and C and Trad A, B and C) and a last cluster which includes the wines produced with the two different methods after 11 months, well separated from the others. The formed clusters suggest that the differences between the two production methods tend to decrease with a longer aging time. Therefore, it appears that the aging process itself influences more the aromatic profile than the production method. The results obtained here are in perfect agreement with what was observed in the analytical analysis of the major volatile compounds. Hence, carbonylic compounds showed marked differences between Charmat and traditional methods at 5 months, but their concentrations reached similar values after 11 months. Similarly, a great difference in the concentration of alcohol was observed at 5 months (with the lower concentrations in the traditional wine) but these differences were no longer significant at 11 months. Moreover, 1‐propanol and ethyl esters increased over time in both wines, reaching a similar amount at 11 months, suggesting that the aromatic profiles tend to be more similar based on the increase in the aging time.

Figure 3 shows the variables of importance in projection (VIPs) responsible for the segregation of the sample scores. Specifically, the QMB 4, 5, 9, 11 and 12, functionalized by Zn, Fe, free base (H3), P and Mn complexes of TPC, respectively, have a VIP value higher than 1 and therefore contribute the most to the clustering of the wine samples. The statistical response of the PLS‐DA model is represented by the confusion matrix (Table 3). The five classes are identified with a variable accuracy of 100% in calibration and between 100% and 80% in cross‐validation. The results indicate that the model perfectly discriminates the different wines. Other studies have found that quartz microbalances can discriminate wines obtained under similar conditions in a protected denomination of origin^23^ or monitor changes in the volatile profile of grapes during their dehydration process.^28^ Moreover, others have demonstrated the ability of QMB‐based E‐noses to follow changes in the aroma profile during wine aging or storage.^16^, ^36^ The production method from which sparkling wine is produced is surely responsible for the main aromatic characteristics. In addition, the aging time is responsible for distinct flavors. The similarity between the e‐nose measurement and analytical data suggests that the e‐nose is able to detect volatile compounds, thus representing a valuable tool for controlling changes that occur in wine bouquets, as aging continues over time.

**Table 3:** Confusion matrix of the PLS‐DA shown in Fig. 2

Confusion matrix	*N*	TPR	FPR	TNR	FNR	Err	P	F1
Base wine	3	1	0	1	0	0	1	1
Charmat 5 months	3	1	0	1	0	0	1	1
Traditional 5 months	3	1	0	1	0	0	1	1
Charmat 11 months	3	1	0	1	0	0	1	1
Traditional 11 months	3	1	0	1	0	0	1	1

Confusion matrix (CV)	*N*	TPR	FPR	TNR	FNR	Err	P	F1
Base wine	3	1	0	1	0	0	1	1
Charmat 5 months	3	1	0	1	0	0	1	1
Traditional 5 months	3	0.6	0	1	0.33	0.06	1	0.80
Charmat 11 months	3	1	0	1	0	0	1	1
Traditional 11 months	3	1	0.08	0.91	0	0.06	0.75	0.85

Err, total error; FNR, false negative ratio; FPR, false positive ratio; F1, F1‐score; *N*, number of classes; P, precision = total positive (TP)/total positive + false positive; TNR, true negative ratio; TPR, true positive ratio.

Principal components regression is a multivariate statistical technique used to model relationships between dependent variables (response variables, E‐nose data) and independent variables (predictor variables, major volatiles) when these variables are strongly correlated, and there are issues with multicollinearity among them. A principal components regression analysis helps to understand the relationships between variables. This technique is particularly useful to verify the ability of E‐nose to predict the amount of the single volatiles identified. As previously mentioned, for the construction of the principal components regression model, three principal components were selected to reach a cumulative explained variability of 96.76% (PC1 87.12, PC2 7.25 and PC3 2.39). The prediction correlation coefficients (*R* ^2^) as well as the root mean square error (RMSEC) of the model are shown in Table 4. Specifically, the highest correlation coefficient (*R* ^2^) was observed for ethanol and volatile acidity (*R* ^2^ of 0.84 and 0.89 in calibration and 0.63 and 0.64 in cross‐validation, respectively). A good prediction ability was also obtained for 1,1‐diethoxyethane and 3‐methyl‐1‐butanol. On the other hand, the worst performance (with an *R* ^2^ below 0.2 in calibration) was observed in the prediction of acetoin and acetaldehyde. Estimated values were not always close to the real values. The model could not quantitatively predict the concentrations of about half of the volatiles identified. However, a good prediction can be performed with this model for some important volatile such as ethanol and volatile acidity. It has to be highlighted that the present model has been constructed with quite a low number of samples. Therefore, it could be improved by analyzing more samples in the same conditions to add more data and improve prediction ability.

**Table 4:** Statistics results of the principal components regression model

Compound	Mean	*N*	Min	Max	RMSEC	RMSECV	*R* ^2^	*R* ^2^ CV
Acetaldehyde	124.68	15	91.83	152.01	17.12	22.73	0.18	0.09
Ethyl acetate	72.71	15	41.21	105.42	13.71	15.96	0.43	0.27
Diethoxyethane	4.09	15	0	21.42	4.17	5.89	0.74	0.49
Methanol	102.01	15	56.71	130.38	18.81	26.32	0.39	0.03
1‐Propanol	41.06	15	37.27	45.86	1.94	2.73	0.42	0.07
Isobutanol	38.22	15	34.24	40.55	0.98	1.43	0.64	0.33
2‐methyl‐1‐butanol	48.62	15	44.66	51.84	1.28	1.75	0.55	0.28
3‐methyl‐1‐butanol	292.76	15	262.55	308.64	6.84	9.07	0.74	0.56
Acetoin	73.36	15	59.66	104.91	12.87	14.90	0.07	0.16
Ethyl lactate	117.93	15	47.43	157.83	27.93	38.13	0.43	0.07
2,3‐Butanediol (*levo*)	542.64	15	322.24	779.83	127.76	161.71	0.29	0.00
2,3‐Butanediol (*meso*)	179.29	15	61.46	260.60	55.17	69.33	0.32	0.02
Diethyl succinate	8.31	15	5.37	11.25	1.34	1.79	0.35	0.02
2‐Phenyl‐ethanol	45.43	15	37.33	55.69	3.69	4.72	0.61	0.37
Ethanol	11.78	15	10	12.5	0.35	0.55	0.84	0.63
Volatile acidity	0.56	15	0.31	0.69	0.04	0.08	0.89	0.64

The mean of three replicates with the minimum and maximum values indicated in the minimum (Min) and maximum (Max) column. *N* represents the number of classes.

RMSEC, root mean square error in calibration; RMSECV, root mean square error in cross‐validation. *R* ^2^, correlation coefficient in calibration. *R* ^2^, correlation coefficient in cross‐validation.

## CONCLUSIONS

The traditional and Charmat methods of making rosé sparkling wine result in different aroma profiles in the final product. This is particularly evident in several of their major volatile compounds. However, over time, even though the wine from the traditional method is still aging on the yeast lees and the Charmat method has already been bottled without lees for storage, the evolution of the wines tends to exhibit fewer differences. Consequently, the most significant variations in the volatile compounds production may occur during the second fermentation within sealed containers. To determine the amounts of volatile compounds, predictive models have been established using the results obtained from GC‐FID and electronic nose analysis. However, most of the estimated concentrations are far from the measured ones. The quantitative prediction could be performed only for a few, but important, compounds (i.e. ethanol and volatile acidity). Overall, the complete volatiles description was not possible through e‐nose. However, considering the excellent discrimination obtained by the PLS‐DA and the similarity of sample clusterization and analytical data, it can be considered a valuable tool for identifying sparkling wines produced with different methods and at different aging times. To validate this methodology on a broader scale, additional experiments involving a wider range of sparkling wines (e.g. from with diverse base wines, yeasts and fermentation conditions) are necessary.

## Data Availability

The data that support the findings of this study are available from the corresponding author upon reasonable request.
